# Cerebrovascular Responsiveness to Hypercapnia Is Stable over Six Months in Older Adults

**DOI:** 10.1371/journal.pone.0143059

**Published:** 2015-11-23

**Authors:** Matthew D. Spencer, Amanda V. Tyndall, Margie H. Davenport, Laurie Argourd, Todd J. Anderson, Gail A. Eskes, Christine M. Friedenreich, David B. Hogan, Richard Leigh, Bernard Meshi, Eric E. Smith, Ben J. Wilson, Stephen B. Wilton, Marc J. Poulin

**Affiliations:** 1 Department of Physiology & Pharmacology, Cumming School of Medicine, University of Calgary, Calgary, Alberta, T2N 4N1 Canada; 2 Hotchkiss Brain Institute, Cumming School of Medicine, University of Calgary, Calgary, Alberta, T2N 4N1 Canada; 3 Libin Cardiovascular Institute of Alberta, Cumming School of Medicine, University of Calgary, Calgary, Alberta, T2N 4N1 Canada; 4 Department of Cardiac Science, Cumming School of Medicine, University of Calgary, Calgary, Alberta, T2N 4N1 Canada; 5 Department of Psychiatry, Faculty of Medicine, Dalhousie University, Halifax, Nova Scotia, B3H 2E2 Canada; 6 Department of Cancer Epidemiology and Prevention Research, Alberta Health Services, Cancer Control Alberta, Calgary, Alberta, T2S 3C3 Canada; 7 Department of Community Health Sciences, Cumming School of Medicine, University of Calgary, Calgary, Alberta, T2N 4N1 Canada; 8 Department of Oncology, Cumming School of Medicine, University of Calgary, Calgary, Alberta, T2N 4N1 Canada; 9 Department of Medicine, Cumming School of Medicine, University of Calgary, Calgary, Alberta, T2N 4N1 Canada; 10 Department of Clinical Neurosciences, Cumming School of Medicine, University of Calgary, Calgary, Alberta, T2N 4N1 Canada; 11 Snyder Institute for Chronic Diseases, Cumming School of Medicine, University of Calgary, Calgary Alberta, T2N 4N1 Canada; 12 Faculty of Kinesiology, University of Calgary, Calgary, Alberta, T2N 1N4 Canada; Hangzhou Normal University, CHINA

## Abstract

The primary purpose of this Brain in Motion (BIM) sub-study was to determine the 6-month stability of resting blood flow velocity and cerebrovascular responsiveness to a euoxic hypercapnic challenge in a group of physically inactive community dwelling men and men aged ≥55 yrs (range 55–92 yrs). At baseline and 6 months later 88 women (65±6 yr) and 78 men (67±7 yr) completed a hypercapnic challenge (step changes from resting end-tidal P_CO2_ ((PET_CO2_) to +1, +5 and +8 mmHg above rest) while cerebral blood flow velocity was assessed using transcranial Doppler ultrasound. Peak velocity of the middle cerebral artery (MCAv) was increased (p<0.05) at the second visit during rest (51±2 vs. 52±4); however, these differences were abolished (p>0.05) when MCAv was normalized to PET_CO2_. During hypercapnia, MCAv tended to be increased at follow-up, but this finding was absent when MCAv/PET_CO2_ was compared across time. Cerebrovascular reactivity (i.e., ΔMCAv/ΔPET_CO2_) was similar (p>0.05) between testing occasions regardless of the approach taken (i.e., considering only the lower step [from +1 to +5 mmHg]; the upper step [+5 to +8 mmHg]; or the complete test taken together). In conclusion, this study has shown that cerebral blood flow and cerebrovascular responsiveness to acute euoxic hypercapnia are stable in older, healthy adults over a 6-month period. Modest changes in MCAv over time must be viewed in the context of underlying differences in PET_CO2_, an important finding with implications for future studies considering cerebral blood flow velocity.

## Introduction

The coming decades will see an exponential growth in the proportion of the population over age 65 years in economically developed countries. Aging in humans, even in the absence of disease, is associated with declines in cardiovascular function. Within the cerebral vasculature, there are reductions in resting blood flow [1] and cerebrovascular responsiveness (i.e., the ability of blood vessels to respond to a stimuli) in some [2–4] but not all [5] studies. As these age-associated changes can be reversed by exercise, they are viewed as potentially crucial links between physical fitness and cognition in older adults [6]. When considering the association between cerebrovascular health and cognitive performance, it is important to consider both blood flow (a proxy for oxygen delivery) under resting conditions and the capacity of the vessel to respond to increased demand under conditions of physiological stress. A number of input stimuli (e.g., hypoxia, hypercapnia) can evoke a vasomotor response within the cerebral vasculature [7–9]. However, the “dose”-response relationship is not necessarily linear across the entire range of physiological values. Both experimental protocols as well as data analysis strategies may potentially influence the reported outcomes.

In light of the association between declines in cognitive performance and post-maturational aging [6,10], considerable research attention is being focused on identifying strategies that successfully attenuate, or possibly reverse, these declines. Notably, improvements in cardiovascular fitness attained through exercise training have been shown to improve both cerebrovascular health and cognitive function in older adults [11–13]. Furthermore, cerebrovascular reserve (e.g., assessment of vascular responsiveness to hypercapnia) is positively associated with better overall cognitive function [6] and has been shown to predict cognitive decline in patients with mild cognitive impairment [14] and is correlated to cortical integrity [15].

Our laboratory has undertaken a combined quasi-experimental and prospective cohort study, called *Brain in Motion* (BIM), to examine the effects of a 6-months aerobic exercise intervention on cerebral blood flow, cerebrovascular responsiveness, and cognitive performance in a group of previously inactive community-dwelling older adults [16]. A major strength of the BIM study design was the inclusion of a 6-month run-in phase, during which participants were instructed to maintain current physical activity levels and lifestyle. During this phase they were not exposed to the exercise intervention portion of the BIM study. This design permitted the determination of the stability of a number of physiological and cognitive variables over the same time period of the intervention phase. The purpose of this paper is to report on the evaluation of the stability of the physiological variables in the pre-intervention phase, as outlined below.

Whereas Strohm et al. [17] have demonstrated that cerebrovascular reactivity to hypercapnia is repeatable day-to-day, at present the stability of cerebral blood flow velocity and cerebrovascular responsiveness over a 6-month period (i.e., a commonly used time frame in interventional studies of cognitive function and aging) have not been established in older adults. Furthermore, the relationship between resting peak cerebral blood flow velocity and cerebrovascular responsiveness is complex [18] and shows evidence of nonlinearity [19,20]–both of which may have important implications with respect to cognitive performance–that has not been described in older adults. The primary purpose of the present study was to determine the 6-month stability of resting cerebral blood flow velocity and cerebrovascular responsiveness in the first 166 BIM participants, a group of healthy, inactive women and men aged ≥55 years, prior to their exposure to the exercise intervention. A secondary purpose was to compare estimates of cerebrovascular responsiveness using a variety of analysis strategies, based on a multi-step and physiologically-relevant hypercapnic challenge. The findings of the present study are relevant for the design of future studies of short-term interventions aimed at improving cerebrovascular functioning and cognitive performance.

## Methods

The BIM study was designed to determine whether a structured 6-month aerobic exercise program would be associated with improvements or maintenance of both cerebrovascular function and cognitive abilities in older individuals and the extent to which any changes observed persist 6 months after the completion of the structured exercise program. The study consists of three sequential six-month phases: 1) pre-intervention; 2) aerobic exercise intervention; and 3) post-intervention [16]. Herein, we are reporting data from a subset of the data from the pre-intervention phase. This sub-study of BIM used data collected during the pre-exercise intervention phase and was designed to address the previously noted study objectives.

### 2.1 Participants

The study population included generally healthy, inactive women and men aged ≥55 years who were cognitively intact and without active, clinically evident cardiorespiratory disease. All women were postmenopausal. Participants were volunteers recruited using posters and newspaper advertisements. All participants provided informed written consent prior to enrollment into the study. The study was approved by the University of Calgary Conjoint Health Research Ethics Board. Specific eligibility criteria were as follows:

### 2.2 Eligibility Criteria

Individuals were considered eligible if they: 1) participated in less than 30 minutes of moderate exercise four days per week or 20 continuous minutes of vigorous exercise two days per week; 2) had a body mass index (BMI) of less than 37.0 kg/m^2^; 3) were able to walk independently outside and up and down at least 20 stairs; 4) had not been diagnosed with overt cardiovascular/cerebrovascular or pulmonary disease; 5) had been non-smokers for at least 12 months; 6) had not undergone major surgery or trauma in the last 6 months; 7) were free of central nervous system disorders such as Multiple Sclerosis; 8) did not have significant cognitive impairment (i.e., Montreal Cognitive Assessment score ≥ 24) [21]; and, 9) had written agreement from their attending physician that there were no contraindications to their participation in the study.

On average the baseline and 6-month testing sessions during the pre-exercise intervention phase were 6.4±1.9 months apart.

### 2.3 Cardiovascular Fitness

Following standard anthropometric measurement of body mass and height, participants completed an incremental exercise test to the limit of tolerance to determine maximal O_2_ uptake (VO_2max_). The incremental exercise tests were conducted on a motorized treadmill and followed a modified Bruce protocol as previously described [16,22]. Briefly, the treadmill speed and grade were progressively increased until the subject could no longer tolerate the exercise intensity. Attainment of VO_2max_ was evidenced by a plateau (<2mL/kg/min) in VO_2_ despite further increases in work rate, a respiratory exchange ratio (RER) of at least 1.15, and/or attainment of age-predicted maximal heart rate (210-(age*0.65)) as recommended by the Canadian Society for Exercise Physiology [23] and American Thoracic Society [24]. VO_2_ and heart rate (HR) were monitored continuously throughout the exercise tests using a metabolic cart and 12-lead ECG, respectively.

### 2.4 Cerebrovascular Function

Trained staff and/or students performed all the testing. Standardized approaches to both fixing the probe and optimizing the angle and depth of insonation were used [25]. The same settings, using standardized landmarking techniques used in our laboratory, were used on each individual for the baseline and 6 Month assessments. Prior to the testing of cerebrovascular responsiveness to CO_2_ participants refrained from eating or drinking anything other than water for two hours prior to testing and also refrained from engaging in exercise on the day of testing. A 2-MHz transcranial Doppler ultrasound (TCD; Toc Neurovision™, Multigon Industries, Inc., Yonkers, NY) was used to measure the blood flow velocity of the middle cerebral artery (MCAv) non-invasively. To locate the MCA, the TCD probe was placed in the temporal region just above the end of the zygomatic process close to the ear, using techniques previously described [7,26,27]. Maximum peak MCA velocity (MCAv), HR (3-lead ECG; Micromon 7142 B, Kontron Medical, Milton Keynes, UK), blood pressure (beat-by-beat using finger pulse photoplethysmography, Finometer, Finapres Medical Systems, Amsterdam, The Netherlands; corroborated with three resting brachial measurements), and arterial O_2_ saturation (finger pulse oximetry; 3900p, Datex-Ohmeda, Madison, WI, USA) were measured continuously throughout the experimental protocol, as previously described [25].

**Fig 1:**
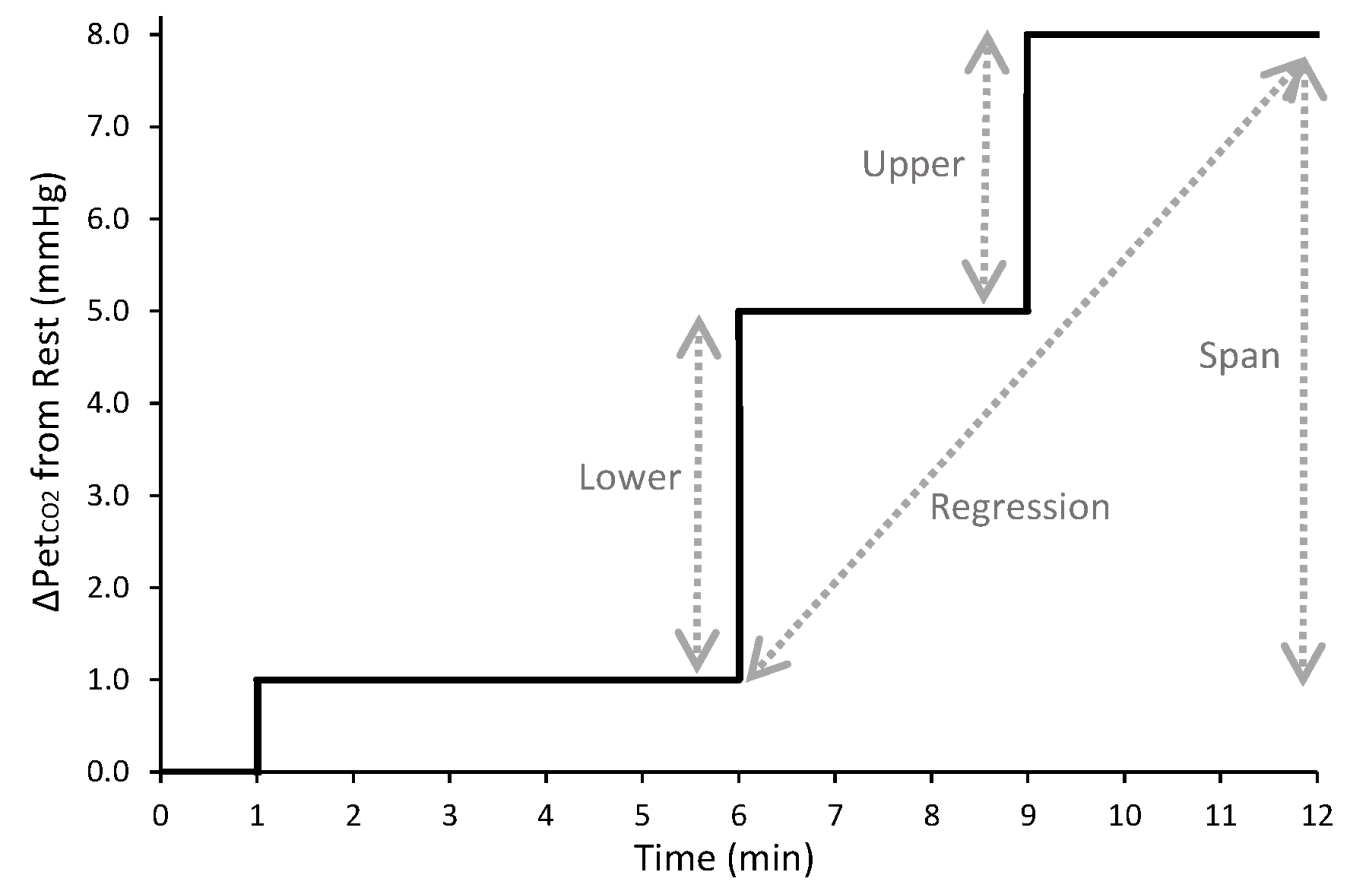
Schematic depicting the hypercapnic challenge completed by subjects at Baseline and 6-month follow-up. Cerebrovascular reserve was calculated using four distinct approaches: “Lower” considered the Δ$\bar{\text{V}}$P/ΔPET_CO2_ during the +1.0 to +5.0 mmHg step change in PET_CO2_; “Upper” considered the Δ$\bar{\text{V}}$P/ΔPET_CO2_ during the +5.0 to +8.0 mmHg step change in PET_CO2_; “Span” considered the total Δ$\bar{\text{V}}$P/ΔPET_CO2_, where Δ was calculated as the difference between +8.0 mmHg and +1.0 mmHg values; and “Regression” calculated the slope of the response across the three stages.

End-tidal P_CO2_ and P_O2_ (PET_CO2_ and PET_O2_) were recorded using dedicated software (Chamber, University Laboratory of Physiology, Oxford, UK) during 10 minutes of seated rest; MCAv, HR, and arterial blood pressures and O_2_ saturation were also monitored continuously throughout this period. With their nose occluded, each subject breathed room air through a mouthpiece connected (via a fine capillary line) to a mass spectrometer (AMIS 2000, Innovision, Odense, Denmark) in which CO_2_ and O_2_ concentrations were analyzed, allowing for determination of PET_CO2_ and PET_O2_, respectively. These end-tidal responses were averaged over the 10 minutes and were used to determine the desired PET_CO2_ and PET_O2_ to assess the cerebrovascular response to a euoxic hypercapnia test. Precise control of desired PET_CO2_ and PET_O2_ values was achieved continuously using customized software (BreatheM v2.40, University Laboratory of Physiology, Oxford, UK) and dynamic end-tidal forcing technique as previously described [7,8,27]. The test, which lasted 12 minutes in total, comprised two 3-minute near-step increases in PET_CO2_. For the first minute, subjects breathed room air only; this was followed by a 5-minute period during which PET_CO2_ was held constant at +1.0 mmHg above the subjects’ 10-minute resting PET_CO2_ value. Using a near-step change, PET_CO2_ was then increased to +5.0 mmHg above normal resting values and held for three minutes and then was further increased to +8.0 mmHg above resting values and held for an additional three minutes (see Fig 1). Physiological responses during the hypercapnic challenge were calculated as the mean response over the final 30 s of each stage (i.e., +1.0, +5.0 and +8.0 mmHg).

Whereas the CVR (cerebrovascular resistance, given as MAP/MCAv (i.e., altered vascular tone primarily affects pressure) [28]) and CVC (cerebrovascular conductance, given as MCAv/MAP (i.e., changes in vascular tone result mainly in changes in blood flow rather than pressure) [28]) describe the “static” conditions within the cerebrovasculature at each PET_CO2_, the sensitivity of the vessels in response to an imposed challenge can be assessed by scaling the absolute change in MCAv (ΔMCAv) to the absolute change in PET_CO2_ (ΔPET_CO2_; input stimulus). Thus, the cerebrovascular responsiveness is given as: ΔMCAv/ΔPET_CO2_. However, the experimental protocol used in the present study allows for this responsiveness to be assessed in a variety of ways. Therefore, cerebrovascular responsiveness was calculated using four distinct approaches (Fig 1): i) the ΔMCAv/ΔPET_CO2_ in response to an initial change in PET_CO2_ (i.e., from +1.0 to +5.0 mmHg; termed “Lower”); ii) the ΔMCAv/ΔPET_CO2_ in response to a subsequent change in PET_CO2_ (i.e., from +5.0 to +8.0 mmHg; termed “Upper”); iii) the ΔMCAv/ΔPET_CO2_ derived by considering the total change across both steps (i.e., where Δ = the difference between +1.0 and +8.0 mmHg; termed “Span”), and using a linear regression to characterize the overall change as the slope of the ΔMCAv—ΔPET_CO2_ relationship across both steps (i.e., considering +1.0, +5.0 and +8.0 mmHg; termed “Regression”).

### 2.5 Statistical Analysis

Descriptive characteristics were estimated and compared between men and women using independent sample t-tests. The within-subjects design of the present study demanded the use of one-way (Time), two-way (Time * Sex), and three-way (Time * Sex * PET_CO2_) repeated measures analyses of variance (ANOVA) to determine statistical significance for the dependent variables. Tukey’s post-hoc analyses were used when significant differences were found for the main effects or interactions of dependent variables. Intraclass correlation coefficients (ICC) were determined to assess the absolute agreement between paired values; the coefficient of variation (CoV) was calculated as the standard deviation of the difference between paired values divided by their mean and divided by √2 [29]. Pearson’s product-moment correlation coefficients were determined to assess the strength of relationships between variables (i.e., non-paired values). All statistical analyses were performed using SPSS Version 20.0, (SPSS Inc., Chicago, IL). All statistical testing was two-sided and statistical significance was defined as p<0.05.

## Results

**Fig 2:**
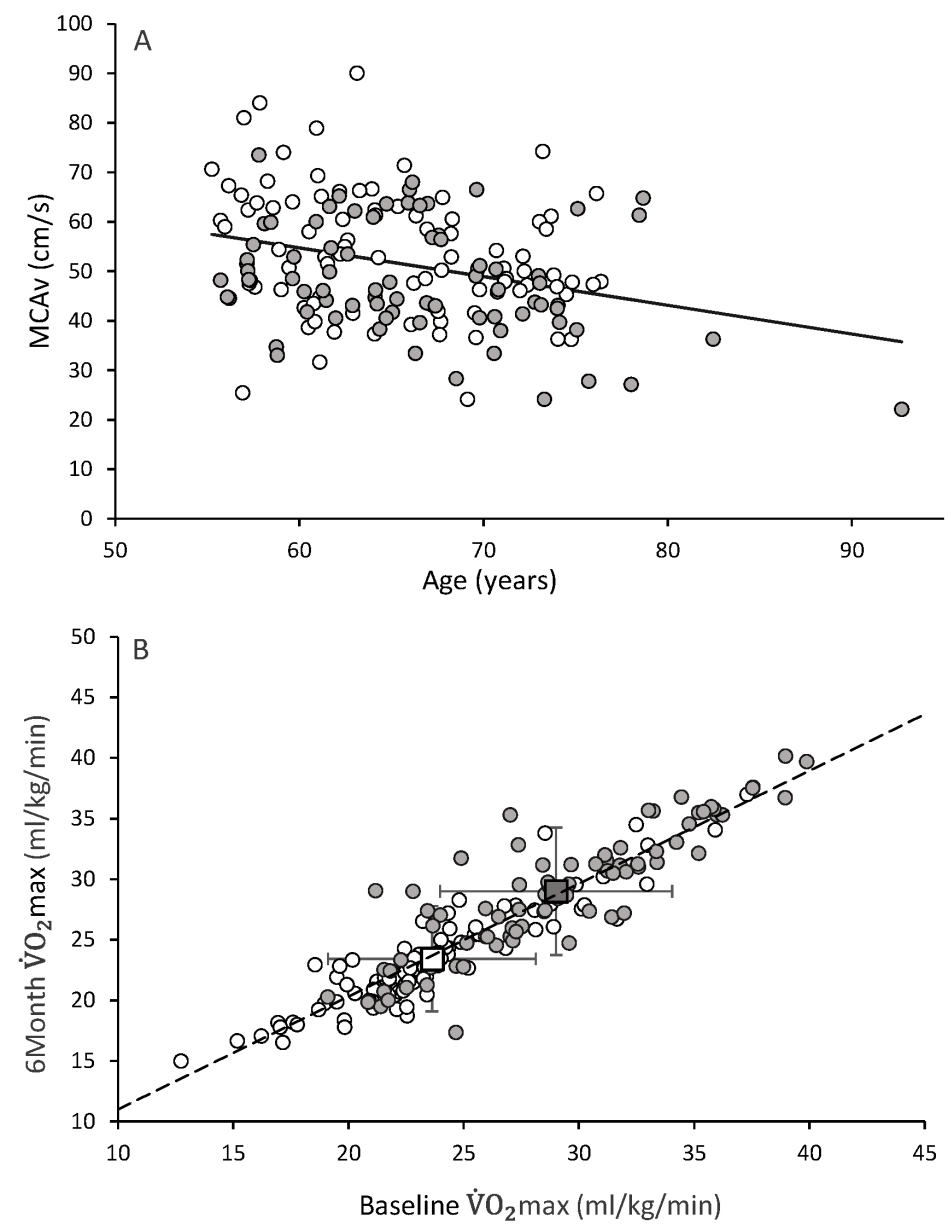
(A) Negative relationship between resting maximal peak cerebral blood flow velocity (MCAv) and age in men and women at Baseline. (B) Stable relationship of subjects’ cardiovascular fitness (VO_2max_) values between Baseline and 6 Months.

The study sample consisted of 88 women (65±6 yr) and 78 men (66±7 yr). At Baseline, men were significantly (p<0.05) taller and heavier than women with similar results observed in these variables at 6 Months (Table 1). Resting MCAv was lower in older participants (Fig 2) at Baseline. Fig 2 confirms that subjects’ cardiovascular fitness (VO_2max_) levels did not change during the 6-month observation period.

**Table 1:** Anthropometric measures, resting heart rate, cerebral blood flow velocity, end-tidal partial pressures for CO_2_ and O_2_, and blood pressures at Baseline and 6 Months.

	Women (n = 88)				Men (n = 78)				All (n = 166)			
	Baseline	6 Month	CoV (%)	ICC	Baseline	6 Month	CoV (%)	ICC	Baseline	6 Month	CoV (%)	ICC
**Height (cm)**	162±6	162±6	0.3	0.998	177±7	177±7	0.2	0.998	169±10	169±10	0.2	0.999
**Mass (kg)**	71±12	70±11	3.2	0.980	87±12	87±12	2.0	0.989	78±14	78±14	2.6	0.990
**BMI (kg/m** ^**2**^ **)**	26.8±4.1	26.6±3.9	3.0	0.979	27.8±3.2	27.7±3.3	2.1	0.983	27.2±3.7	27.1±3.7*	2.6	0.981
**HR (bpm)**	66±9	67±8	9.1	0.668	64±8	64±10	9.1	0.764	65±9	66±9	9.1	0.727
**MCAv (cm∙s** ^**-1**^ **)**	53.9±12.7	54.8±11.6	9.7	0.895	48.1±11.2	49.7±11.0*	9.6	0.896	51.2±12.3	52.4±11.5*	9.7	0.901
**PET**_**CO2**_	35.4±3.5	36.2±3.5*	5.6	0.795	35.0±3.0	35.5±3.0	5.5	0.736	35.2±3.2	35.9±3.3*	5.5	0.773
**PET**_**O2**_	85.7±4.8	85.5±4.6	3.6	0.738	86.2±4.0	86.7±4.6	3.8	0.574	86.0±4.4	86.0±4.6	3.7	0.674
**SBP**	127±19	125±19	8.8	0.797	131±16	128±14	9.0	0.601	129±18	126±17*	8.9	0.733
**DBP**	73±12	72±9	10.8	0.659	78±9	76±11	8.1	0.768	75±11	74±11*	9.5	0.723
**MAP**	91±13	89±11	8.9	0.727	95±10	93±11	7.4	0.703	93±12	91±11*	8.2	0.728

Values are mean±SD; units are mmHg except where indicated. BMI, body mass index; HR, heart rate; MCAv, maximum peak blood flow velocity in the middle cerebral artery; PET_CO2_, end-tidal partial pressure for CO_2_; PET_O2_, end-tidal partial pressure for O_2_; SBP, systolic blood pressure; DBP, diastolic blood pressure; MAP, mean arterial pressure; CoV, coefficient of variation; ICC, intraclass correlation coefficient.

*, p<0.05 from Baseline.

### 3.1 Resting Measures

The resting HR, MCAv, PET_CO2_ and PET_O2_, and blood pressures recorded at both Baseline and 6 Months are shown in Table 1. Resting MCAv and PET_CO2_ were both significantly greater (p<0.05) at 6 Months (Table 1). In addition, systolic, diastolic and mean blood pressures were slightly but statistically significantly lower at 6 Months.

### 3.2 Cerebral Blood Flow Responses during Hypercapnic Challenge

In contrast to the resting condition, MCAv was not increased at 6 Months compared with Baseline at any of +1.0 mmHg, +5.0 mmHg and +8.0 mmHg, respectively (Table 2).

**Table 2:** Cerebrovascular responses during hypercapnic challenge at Baseline and 6 Months.

	Women (n = 88)				Men (n = 78)				All (n = 166)			
	Baseline	6 Month	CoV (%)	ICC	Baseline	6 Month	CoV (%)	ICC	Baseline	6 Month	CoV (%)	ICC
**+1.0 mmHg**												
HR (bpm)	65±8	66±8	6.5	0.822	63±8	63±10	7.7	0.837	64±8	64±9	7.1	0.834
MAP (mmHg)	89±11	86±11*	7.5	0.760	92±9	91±10	6.6	0.755	90±10	88±11*	7.1	0.766
MCAv (cm∙s^-1^)	55.9±12.9	56.3±11.8	9.7	0.892	48.5±11.4	50.6±11.0*	9.3	0.901	52.4±12.7	53.6±11.7	9.6	0.903
CVR (mmHg/cm∙s^-1^)	1.68±0.47	1.60±0.39*	13.3	0.844	2.01±0.57	1.88±0.46*	12.5	0.859	1.84±0.54	1.73±0.44*	13.0	0.867
CVC (cm∙s^-1^/mmHg)	0.64±0.17	0.67±0.18*	12.0	0.883	0.53±0.14	0.56±0.13*	11.8	0.856	0.59±0.16	0.62±0.16*	11.9	0.887
**+5.0 mmHg**												
HR (bpm)^†^	66±8	67±8	7.2	0.760	64±8	64±10	6.6	0.885	65±8	65±9	7.0	0.836
MAP (mmHg)^†^	93±12	91±12	9.0	0.679	97±11	95±12	7.7	0.718	95±12	93±12*	8.3	0.707
MCAv (cm∙s^-1^)^†^	63.0±15.1	63.5±13.5	10.4	0.884	55.8±13.9	57.2±12.5	9.7	0.905	59.6±15.0	60.6±13.3	10.1	0.899
CVR (mmHg/cm∙s^-1^)^†^	1.57±0.45	1.50±0.37*	14.7	0.822	1.86±0.54	1.73±0.43*	12.1	0.872	1.71±0.51	1.61±0.42*	13.4	0.863
CVC (cm∙s^-1^/mmHg)^†^	0.69±0.19	0.71±0.19	12.2	0.888	0.58±0.16	0.61±0.14*	12.0	0.867	0.64±0.19	0.66±0.18*	12.1	0.891
**+8.0 mmHg**												
HR (bpm)^†^ ^‡^	68±9	69±8	7.5	0.760	65±9	65±11	7.7	0.846	67±9	67±9	7.6	0.814
MAP (mmHg)^†^ ^‡^	99±14	97±14	10.3	0.660	103±12	101±12	7.9	0.701	101±13	99±14*	9.2	0.686
MCAv (cm∙s^-1^)^†^ ^‡^	71.0±17.5	71.9±15.4	11.5	0.858	63.5±16.6	65.3±14.7	10.7	0.891	67.5±17.4	68.8±15.4	11.2	0.879
CVR (mmHg/cm∙s^-1^)^†^ ^‡^	1.49±0.46	1.41±0.36*	16.3	0.800	1.75±0.54	1.63±0.41*	14.2	0.844	1.61±0.52	1.51±0.40*	15.2	0.837
CVC (cm∙s^-1^/mmHg)^†^ ^‡^	0.73±0.21	0.76±0.21*	12.6	0.880	0.62±0.18	0.65±0.16*	12.8	0.864	0.68±0.20	0.71±0.20*	12.7	0.884

Values are mean±SD. HR, heart rate; MAP, mean arterial pressure; MCAv, maximum peak blood flow velocity recorded in the middle cerebral artery; CVR, cerebrovascular resistance (MAP/MCAv); CVC, cerebrovascular conductance (MCAv/MAP); CoV, coefficient of variation; ICC, intraclass correlation coefficient.

*, p<0.05 from Baseline

^†^, p<0.05 from +1.0mmHg

^‡^, p<0.05 from +5.0mmHg.

To examine whether or not these differences in MCAv (at rest) were a reflection of differences in PET_CO2_ between measurement occasions, we calculated the MCAv/PET_CO2_ ratio at rest and for each of +1.0, +5.0 and +8.0 mmHg test conditions. When MCAv was scaled as a function of PET_CO2_, no differences were observed between Baseline and 6 Months at any stage of the hypercapnic challenge (i.e., Rest, +1.0, +5.0 and +8.0 mmHg) (Table 3).

**Table 3:** Middle cerebral artery velocity (MCAv) normalized to PET_CO2_ during hypercapnic challenge at Baseline and 6 Months.

	Women (n = 88)				Men (n = 78)				All (n = 166)			
	Baseline	6 Month	CoV (%)	ICC	Baseline	6 Month	CoV (%)	ICC	Baseline	6 Month	CoV (%)	ICC
**Rest**	1.52±0.34	1.51±0.29	9.3	0.887	1.37±0.30	1.40±0.29	9.5	0.891	1.45±0.33	1.46±0.30	9.4	0.893
**+1.0 mmHg**	1.53±0.33	1.52±0.28	9.5	0.877	1.35±0.29^§^	1.39±0.29	9.6	0.883	1.45±0.33	1.46±0.29	9.6	0.887
**+5.0 mmHg**	1.56±0.35^§^ ^†^	1.54±0.30^§^ ^†^	10.1	0.868	1.39±0.32^†^	1.42±0.29^†^	9.2	0.903	1.48±0.34^§^ ^†^	1.48±0.30^§^ ^†^	9.8	0.890
**+8.0 mmHg**	1.64±0.38^§^ ^†^ ^‡^	1.63±0.32^§^ ^†^ ^‡^	11.4	0.837	1.48±0.36^§^ ^†^ ^‡^	1.51±0.32^§^ ^†^ ^‡^	10.3	0.887	1.56±0.38^§^ ^†^ ^‡^	1.57±0.33^§^ ^†^ ^‡^	11.0	0.866

Values are mean±SD; units are (cm/s)/mmHg; CoV, coefficient of variation; ICC, intraclass correlation coefficient.

^§^, p<0.05 from Rest

^†^, p<0.05 from +1.0 mmHg

^‡^, p<0.05 from +5.0 mmHg.

**Fig 3:**
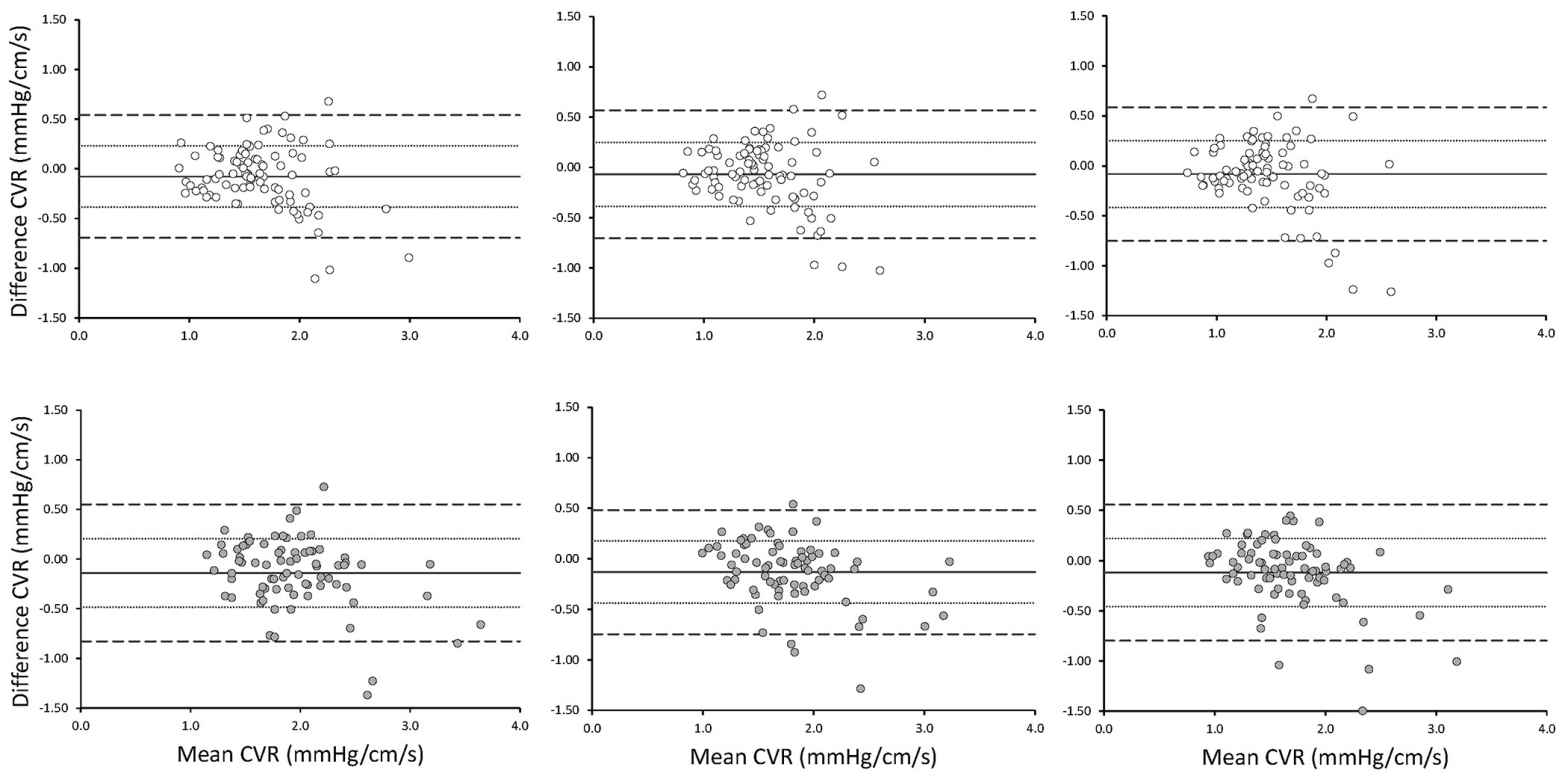
Bland-Altman plots illustrating differences in CVR between Baseline and 6 Month measurements in women (top panels; empty circles) and men (bottom panels; filled circles). Responses observed at +1.0 mmHg (left), +5.0 mmHg (center), and +8.0 mmHg (right) of above resting PET_CO2_ are presented separately.

**Fig 4:**
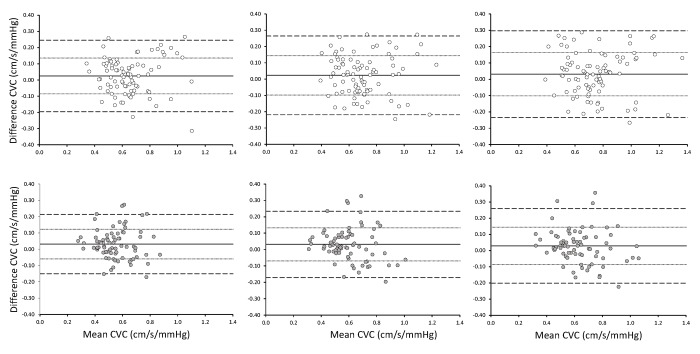
Bland-Altman plots illustrating differences in CVC between Baseline and 6 Month measurements in women (top panels; empty circles) and men (bottom panels; filled circles). Responses observed at +1.0 mmHg (left), +5.0 mmHg (center), and +8.0 mmHg (right) of above resting PET_CO2_ are presented separately.

Figs 3 and 4 illustrate the differences (i.e., bias), precision (mean±SD), and limits of agreement (mean±2SD) between CVR and CVC measures, respectively, at Baseline and 6 Months for each individual for each stage of the hypercapnic challenge [30]. Starting with CVR for women (Fig 3, top panels), the bias and precision values at +1, +5 and +8 mmHg above resting PET_CO2_ were -0.07 (-0.38 to 0.24), -0.07 (-0.39 to 0.25), and -0.08 (-0.42 to 0.25), respectively. For CVR in men (Fig 3, bottom panel), the bias and precision values at +1, +5 and +8 mmHg above resting PET_CO2_ were -0.14 (-0.44 to 0.18), -0.13 (-0.44 to 0.18), and -0.12 (-0.46 to 0.22), respectively. Turning to CVC for women (Fig 4, top panels), the bias and precision values at +1, +5 and +8 mmHg above resting PET_CO2_ were 0.02 (-0.86 to 0.13), 0.02 (-0.10 to 0.14), and 0.03 (-0.10 to 0.16), respectively. For CVC in men (Fig 4, bottom panel), the bias and precision values at +1, +5 and +8 mmHg above resting PET_CO2_ were 0.03 (-0.60 to 0.12), 0.03 (-0.70 to 0.13), and 0.03 (-0.09 to 0.144), respectively.

### 3.3 Cerebrovascular Responsiveness

**Fig 5:**
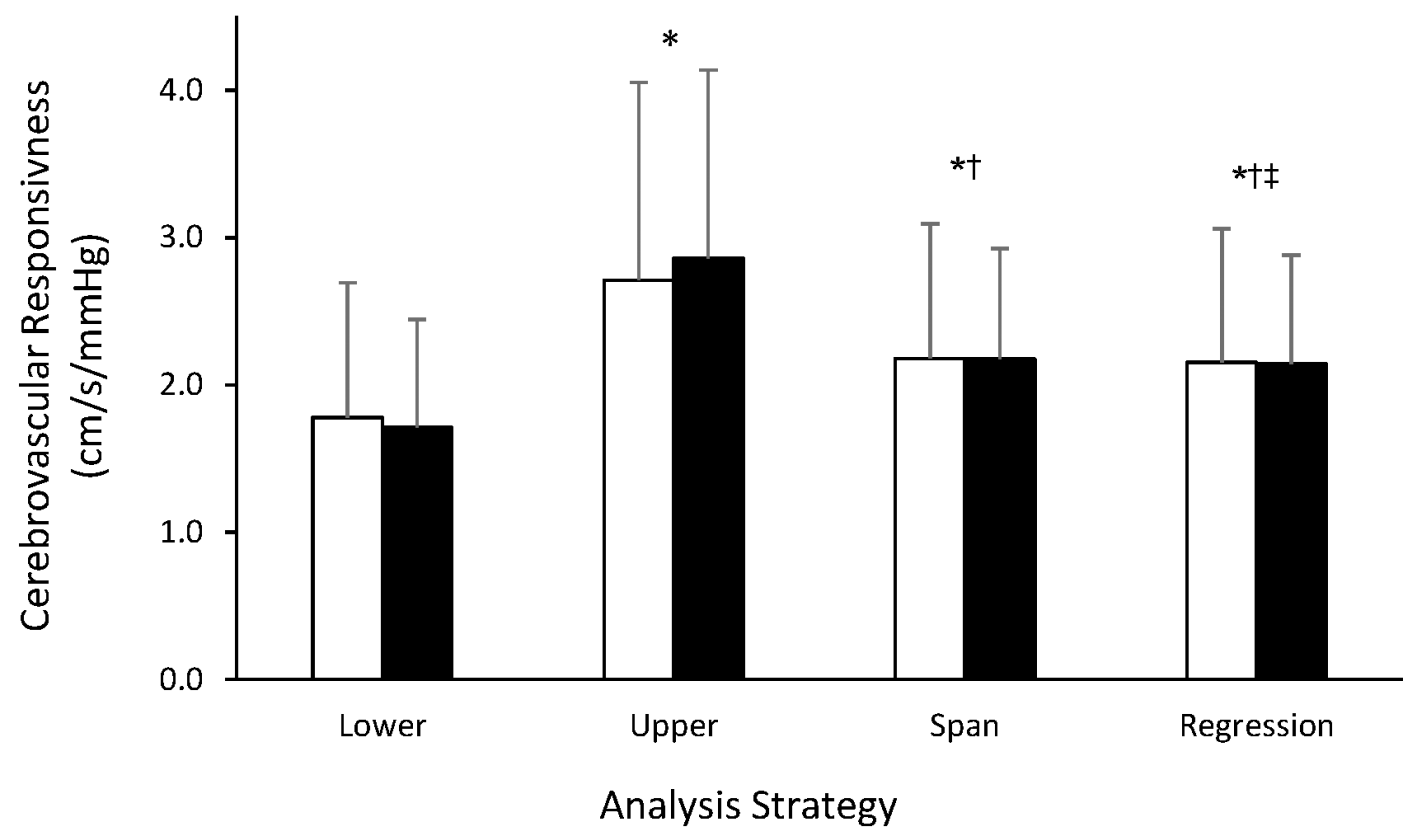
Comparison of strategies for computing cerebrovascular responsiveness in older adults undergoing a hypercapnic challenge. Values are mean±SD. *, p<0.05 from Lower; ^†^, p<0.05 from Upper; ^‡^, p<0.05 from Span.

**Fig 6:**
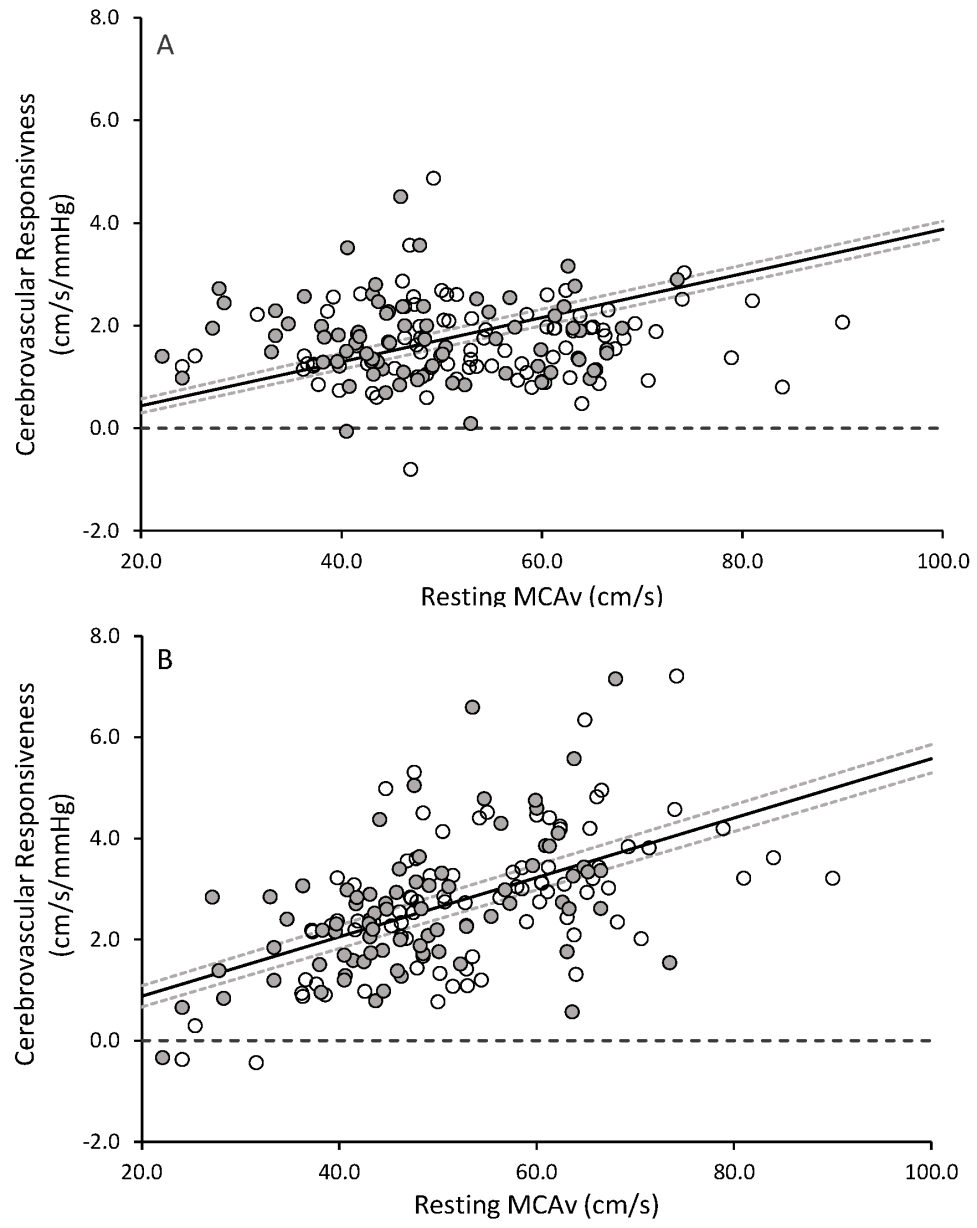
Relationship between resting maximal peak cerebral blood flow velocity (MCAv) and cerebrovascular response to (A) the Lower PET_CO2_ step and (B) the Upper PET_CO2_ step. Data presented are from Baseline testing occasion.

Fig 5 illustrates the cerebrovascular responsiveness at Baseline and 6 Months, and compares the values when computed as the change from +1.0 to +5.0 mmHg (i.e., Lower), +5.0 to +8.0 (i.e., Upper), +1.0 to +8.0 (i.e., Span), and as the linear regression through all three levels of PET_CO2_ (i.e., Regression). Whereas these four analytic strategies all differed (p<0.05) from one another, no differences in cerebrovascular reactivity were detected between Baseline and 6 Months, regardless of the analysis strategy employed. The correlations between Lower and Upper steps (i.e., the only analyses strategies that did not contain overlapping data) were r = 0.33 (p<0.05) and r = 0.28 (p<0.05) for Baseline and 6 Months, respectively. Finally, Fig 6 illustrates that the cerebrovascular responsiveness in response to both Lower and Upper steps, respectively, is associated with resting MCAv.

## Discussion

Our main findings were as follows: i) while resting MCAv and PET_CO2_ were both greater at 6 Months as compared with Baseline, the MCAv/PET_CO2_ ratio was stable over this interval; ii) the MCAv was similar between measurement occasions throughout the hypercapnic challenge, despite a significantly greater PET_CO2_ (secondary to a greater resting PET_CO2_). As was the situation with resting measures, no differences were observed between Baseline and 6 Months when the MCAv/PET_CO2_ ratio was considered; iii) regardless of the analysis strategy used to calculate cerebrovascular responsiveness, no differences were observed between Baseline and 6 Months, though the four strategies examined each yielded a significantly different estimate for responsiveness. These data indicate that cerebrovascular responsiveness is stable over a 6 month period in older women and men, provided that a standardized approach is employed); and, iv) cerebrovascular responsiveness is associated with resting cerebral blood flow velocity in this group of older adults.

With respect to the expected effects of aging (a modest decline in resting MCAv of ~0.30 cm∙s^-1^ over the course of the study), the actual changes observed were in the opposite direction. Two conclusions seem supportable by the data here: i) the effects of aging *per se* likely cannot be assessed longitudinally in periods shorter than several years; and, ii) the influence of factors other than aging (i.e., PET_CO2_) can have dramatic effects on resting MCAv. The present findings reinforce the high degree of dependence of MCAv on PET_CO2_. Future studies in which an intervention is expected to change MCAv (or in control groups wherein MCAv is not expected to change) should consider subjects’ resting and experimental PET_CO2_ when assessing changes in MCAv over time.

Study participants’ body size (i.e., height, body mass, and BMI) and cardiovascular fitness (i.e., VO_2max_) showed no significant change from Baseline to 6 Months. These observations indicated that that our subjects did not modify their lifestyles in a significant manner during this pre-intervention phase of the study. Changes in cerebral blood flow and cerebrovascular responsiveness should not therefore be expected. Under resting conditions, a 1.2 cm∙s^-1^ (~2.3%) increase (p<0.05; Table 1) in MCAv was observed between testing occasions. In spite of the strong intraclass correlations between measures taken at the two testing occasions, this significant difference in resting blood flow velocity between visits may suggest that the measure is not entirely stable in the population tested. This conclusion, however, may be an oversimplification since MCAv is highly sensitive to changes in PET_CO2_, as well as blood pressure, among other variables. When MCAv values were adjusted for resting baseline PET_CO2_ (35.2 *vs* 35.9 mmHg at Baseline and 6 Months, respectively; p<0.05, Table 1), these differences were no longer present. The latter finding also provides good evidence that there was not simply an “operator” effect due to how the assessors fixed the TCD probe and obtained the velocity profile, as such differences could not be accounted for by controlling for differences in PET_CO2_.

Given that the hypercapnic challenge used in the present study required subjects to undergo three “step changes” in PET_CO2_ (i.e., +1.0, +5.0, and +8.0 mmHg) that were initiated from the individually established resting values, it was unexpected that the differences observed at rest did not persist throughout hypercapnia. In aggregate, these findings suggest that MCAv is relatively stable, particularly when considering the impact of underlying differences in PET_CO2_ that could account for the differences observed (at rest). The factors that may cause a change in PET_CO2_ are beyond the scope of the present study, but examination of the ventilatory response during hypercapnia precludes the possibility of a relative hyperventilation at Baseline as compared with 6 Months (which would be expected to reduce PET_CO2_).

At all three hypercapnic stages, differences in CVR (decreased) and CVC (increased) were observed between Baseline and 6 Months. At the same time, differences were observed for mean arterial pressure (MAP) at the three stages. Thus, these differences in CVR and CVC were likely influenced primarily by modest, but statistically significant differences in MAP. It is apparent in Figs 3 and 4 that while the bias (i.e., mean difference) was significantly different from 0, the precision and limit of agreements are relatively narrow.

The ability of the cerebrovasculature to respond to an external stimulus (e.g., step changes in PET_CO2_) has been proposed as a potential link between cardiovascular fitness and cognitive performance in older adults [6]. We assessed responsiveness as the change in MCAv (ΔMCAv) divided by the change in PET_CO2_ (ΔPET_CO2_); however, given the multi-step nature of the testing protocol, four distinct analysis strategies were identified. Although we observed no differences between Baseline and 6 Months for any of the analytic approaches, we found that the four strategies provided four distinct estimates for cerebrovascular responsiveness. Notably, the greatest and smallest estimates were observed during the Upper and Lower steps, respectively. This result suggests an increased sensitivity at higher compared to lower levels of PET_CO2_, and again reinforces the importance of considering resting values. Another conclusion to be drawn from this observation is that given that Lower and Upper steps have different slopes, it may not be appropriate to characterize the overall response using the linear regression. Similarly, the “Span” strategy (i.e., considering the overall change between +1.0 and +8.0 mmHg) is subject to criticism, since subjects did not actually undergo a step change from +1.0 to +8.0 mmHg; that is, the response may have differed had the +5.0 mmHg condition not been included. Whether the +5.0 mmHg step alters the response observed at +8.0 mmHg remains to be determined.

The correlations between the Lower and Upper approaches, while statistically significant, were relatively low (particularly at 6 Months), and thus, appear to provide distinctly different information on cerebrovascular responsiveness. Whereas the conditions imposed in the Lower step are perhaps more likely to be encountered in daily life as compared with the Upper step, there still appears to be value in conducting multiple step changes in PET_CO2_, and analyzing each of them separately. Interestingly, the responsiveness during both the Lower and Upper PET_CO2_ steps were associated with resting MCAv. This novel observation may imply that resting cerebral blood flow velocity can provide insights into vascular function (i.e., reactivity or responsiveness), even in the absence of an imposed stressor.

### 4.1 Limitations

Transcranial Doppler ultrasound is a technique commonly used to measure MCAv as an estimate of cerebral blood flow, but some limitations of the technique should be discussed. The interpretation of transcranial Doppler ultrasound measurements as a tool that reflects relative changes in blood flow relies on the assumption that the diameter of the vessel being insonnated remains constant. Transcranial Doppler ultrasound does not permit the assessment of changes in diameter *per se*, but it is possible to use the total power of the reflected Doppler signal as an index of relative changes in cross sectional area of the vessel being insonated [31]. The theoretical approach of this index of cerebral blood flow has been confirmed in previous studies utilizing flow phantoms [32,33]. In addition, by considering the entire velocity spectrum (*i*.*e*., the intensity weighted mean velocity) a computational index of cerebral blood flow can be calculated as previously described [7,27,31]. The findings from previous studies using this approach [7,31] suggest that during steady-state moderate euoxic hypercapnia (i.e., 7–8 mmHg above near resting end-tidal PCO_2_ levels), the cross sectional area of the middle cerebral artery remains relatively constant and that this approach is suitable and appropriate.

Recent MRI studies have examined the question of middle cerebral artery dilation during manipulations in end-tidal PCO_2_ and inconclusive results were reported [34,35]. In the Coverdale study [34], a 16% increase in the cross-sectional area of the MCA was reported when end-tidal PCO_2_ was raised by 10 mmHg above resting values by enriching an air mixture with 6% CO_2_. In contrast, the Verbee study [35] found no significant changes in vessel diameter in the MCA when end-tidal PCO_2_ was increased by 7.5 mmHg using a manual technique to control end-tidal PCO_2_ at the desired target. The reason for these very different findings in these technically challenging studies is not clear but they likely relate to factors such as MRI protocols and resolution (i.e., 3 [34] *vs* 7 [35] Tesla), magnitude increase and duration of the CO_2_ challenge (7.5–15.0 mmHg above resting values), and variability in the accuracy of end-tidal PCO_2_ control and targeting (fixed inspired [34] vs manual control [35]), which will impact actual arterial PCO_2_ circulating in the brain [36]. Clearly, further work is needed to fully validate MRI-based measurements of middle cerebral artery dilation in response to CO_2_ in humans.

The traditional approach to understanding cerebrovascular regulation describes a model whereby changes in resistance occur at the level of the arteriolar (i.e., pial) vessels. Recent studies suggest that large conduit-type arteries such as the middle cerebral artery may contribute to the regulation of CVR during acute changes in blood gases [37,38]. However, these findings may or may not be consistent with the MRI findings reported above [34,35]. The general consensus using the highest possible resolution MRI [35] and transcranial Doppler ultrasound [27] suggest that during acute exposure to mild to moderate euoxic hypercapnia such as the level used in this study and others [35], the cross-sectional area of large intracranial arteries such as the MCA remains relative constant. Finally, although the stimulus used in this study is modest, we cannot fully dismiss the possibility of a small dilation of the middle cerebral artery that is less than the error of measurement, thereby potentially contributing to a small underestimation in the reported cerebrovascular responses to euoxic hypercapnia. It is also not possible to dismiss the possibility that the changes reported in the middle cerebral artery are not representative of changes in other parts of the brain [37].

We also cannot rule out small but potentially confounding effects of blood pressure changes on cerebrovascular responses to hypercapnia. While the increases in cerebral blood flow with hypercapnia are thought to be due primarily to CO_2_ *per se*, increases in mean arterial blood pressure likely played a small but significant role in the overall vascular response [39–42]. It is also necessary to consider the effects of the response dynamics of the cerebral circulation to hypercapnia, which were described in detail by Poulin et al. [7]. While the duration of the CO_2_ steps was chosen to allow steady-state cerebrovascular responses to unfold at each step, we cannot rule out the possibility that steady-state responses were not fully established at each CO_2_ step. This may have played a role in explaining part of the differences observed between strategies used to assess the cerebrovascular responses, but not the reproducibility of the results, illustrated in Fig 5 [39]. However, since we used the same protocol at Baseline and 6 Months, effects of blood pressure and CO_2_ step timing are unlikely to have affected the bias and precision of our results.

Finally this sub-study did not seek to explore the relationship between resting cerebral blood flow and cognitive decline, or the relationship between cerebrovascular responses to CO_2_ and cognitive decline in older humans. For example, we did not address the question of whether changes in cognitive function over a period of 6-months are associated with changes in resting cerebral blood flow or cerebrovascular responses to CO_2_ over the same time period. All the participants in the current study were physiologically and cognitively healthy, and it is unlikely that large declines in cognitive function would be observed over this short time period. However, in future work as part of our *Brain in Motion* study, we plan to assess the relationship between cerebrovascular responsiveness and cognition in response to an exercise intervention and after a follow-up period [16].

In conclusion, cerebral blood flow velocity and cerebrovascular responsiveness appear to be stable over 6 months, particularly once the unexpected differences in PET_CO2_ are taken into consideration. These findings validate this study design choice of the BIM study, and highlight the importance of considering underlying differences in PET_CO2_ in future studies considering cerebral blood flow velocity. Specifically, the present study has shown that resting, but not hypercapnic MCAv, was greater at 6 Months compared with Baseline assessments in older adults, but that these differences were abolished when differences in PET_CO2_ were accounted for. No sex-differences were observed in these responses. Finally, regardless of the analytic method used to calculate cerebrovascular responsiveness, no differences were observed between Baseline and 6 Months; however, the four strategies (i.e., Lower, Upper, Span, and Regression) examined each yielded a significantly different estimate for responsiveness.
